# Antimicrobial resistance (AMR) nanomachines—mechanisms for fluoroquinolone and glycopeptide recognition, efflux and/or deactivation

**DOI:** 10.1007/s12551-018-0404-9

**Published:** 2018-03-10

**Authors:** Mary K. Phillips-Jones, Stephen E. Harding

**Affiliations:** National Centre for Macromolecular Hydrodynamics, School of Biosciences, University of Nottingham, Sutton Bonington, LE12 5RD Loughborough, Leicestershire UK

**Keywords:** Antimicrobial resistance, Glycopeptide, Fluoroquinolone, Hydrodynamics, Analytical ultracentrifugation, Circular dichroism spectroscopy

## Abstract

In this review, we discuss mechanisms of resistance identified in bacterial agents *Staphylococcus aureus* and the enterococci towards two priority classes of antibiotics—the fluoroquinolones and the glycopeptides. Members of both classes interact with a number of components in the cells of these bacteria, so the cellular targets are also considered. Fluoroquinolone resistance mechanisms include efflux pumps (MepA, NorA, NorB, NorC, MdeA, LmrS or SdrM in *S. aureus* and EfmA or EfrAB in the enterococci) for removal of fluoroquinolone from the intracellular environment of bacterial cells and/or protection of the gyrase and topoisomerase IV target sites in *Enterococcus faecalis* by Qnr-like proteins. Expression of efflux systems is regulated by GntR-like (*S. aureus* NorG), MarR-like (MgrA, MepR) regulators or a two-component signal transduction system (TCS) (*S. aureus* ArlSR). Resistance to the glycopeptide antibiotic teicoplanin occurs via efflux regulated by the TcaR regulator in *S. aureus*. Resistance to vancomycin occurs through modification of the D-Ala-D-Ala target in the cell wall peptidoglycan and removal of high affinity precursors, or by target protection via cell wall thickening. Of the six Van resistance types (VanA-E, VanG), the VanA resistance type is considered in this review, including its regulation by the VanSR TCS. We describe the recent application of biophysical approaches such as the hydrodynamic technique of analytical ultracentrifugation and circular dichroism spectroscopy to identify the possible molecular effector of the VanS receptor that activates expression of the Van resistance genes; both approaches demonstrated that vancomycin interacts with VanS, suggesting that vancomycin itself (or vancomycin with an accessory factor) may be an effector of vancomycin resistance. With 16 and 19 proteins or protein complexes involved in fluoroquinolone and glycopeptide resistances, respectively, and the complexities of bacterial sensing mechanisms that trigger and regulate a wide variety of possible resistance mechanisms, we propose that these antimicrobial resistance mechanisms might be considered complex ‘nanomachines’ that drive survival of bacterial cells in antibiotic environments.

## Introduction

The term ‘*Antimicrobial Resistance (AMR) Nanomachine*’ (or *‘AMR Nanomachine*’) has not to our knowledge been coined previously. Yet it may be considered an appropriate term to use for the cascade of molecular mechanisms that drive antimicrobial drug resistances in microorganisms, including drug recognition by intricate microbial sensing and signal transduction machinery and the subsequent efflux and/or deactivation machinery that remove the antimicrobials death threat from microbial cells. Our definition of the AMR nanomachine therefore encompasses the initiation, activity and control of AMR in response to a given antibiotic in bacterial cells. The AMR nanomachine has one overarching goal—one machinery—aimed at the overall process of achieving resistance to an antibiotic (AMR). The individual processes are not able to function independently of each other if AMR is to be achieved—indeed, they are very much interconnected through the antibiotic to which they are reacting, and dependent on each other to achieve the AMR goal. After all, when antibiotic is removed, all these processes are either reduced or cease to function. The AMR nanomachine can be switched on and off by levels of antibiotic present. Together, these machines facilitate the survival of microorganisms in environments containing elevated levels of antimicrobial drugs. Unfortunately, one such environment includes our hospitals and clinics that utilise antimicrobial agents to combat microbial infections. Possession of the resistance ‘nanomachinery’ by pathogenic microorganisms poses a serious threat to our ability to treat serious microbial infections with current therapies. Indeed, resistance exhibited by bacterial pathogens to current antibacterial agents is now recognised to be a major global problem in the fight against infections. Currently 25,000 people per annum die in Europe as a result of infections caused by microorganisms that are untreatable with antimicrobial agents (EARS-Net 2014; Public Health England Report 2015) and it is predicted that there will be 10 million deaths every year globally by 2050 unless action is taken to safeguard the effectiveness of our antibiotics (HM Government (UK) Review 2015). Antibiotic-resistant infections are also estimated to cost the European Union €1.5 billion per year with regard to healthcare expenses and lost productivity; by 2050, costs worldwide are predicted to soar to £66 trillion (Public Health England Report 2015; HM Government (UK) Review 2015).

Major causes of the emergence and development of resistance machines amongst microbial populations are the intense use and misuse of antibiotics (reviewed in Barbosa and Levy 2000). The more that antibiotics are used and distributed in the environment, the greater the generation of multi-antibiotic resistances (e.g. Mladenovic-Antic et al. 2016; Tammer et al. 2016; Barnes et al. 2017; Mascarello et al. 2017; Pitiriga et al. 2017; also see CMO Report 2011; Public Health England and Veterinary Medicines Directorate Report 2015). After all, resistance can be considered a natural phenomenon and, as already mentioned above, a means by which microorganisms protect themselves against exposure to antibiotics in the environment. In the UK human healthcare sector, 531 tonnes of active antibiotics were prescribed in 2013 (Public Health England Report 2013). In spite of high usage, the importance of rational use of antibiotics has been highlighted previously (Aliabadi and Lees 2000). Dosing regimens and durations of antibiotic treatments should be optimised so that they are sufficiently high as to maximise antibacterial effect but as low as possible to reduce the risk of the emergence of resistance (Baquero and Negri 1997; Guillemot et al. 1998; Negri et al. 1994). The use of sub-optimal antibiotic dosages, as well as excessive dosages, increase selection of resistant strains (Odenholt et al. 2003; Baquero et al. 2008; Gullberg et al. 2011); mathematical modelling methods are being explored to investigate optimal doses and durations (e.g. Bonhoeffer et al. 1997; Bergstrom et al. 2004; D’Agata et al. 2008; Geli et al. 2012; Peña-Miller et al. 2014; Paterson et al. 2016).

In an era in which fewer new and novel antibiotics (that might overcome the resistance issue) are being discovered, the Chief Medical Officer for England has called for antimicrobial stewardship measures to be put in place, encompassing the promotion and monitoring of the judicious use of existing antimicrobials to preserve their future effectiveness (CMO Report 2011). Two of the most important classes of antibiotics recognised as critically important to both medicine and agriculture are the fluoroquinolones and the glycopeptides (WHO 2011; OiE 2015). These antibiotic classes are considered of utmost priority with regard to risk management of resistance generation amongst microbial populations (WHO 2011; OiE 2015). This review will focus on these priority classes, describing the fluoroquinolone and glycopeptide resistance machinery found in enterococci and staphylococci (bacteria that are of significance (and common) to both animal husbandry and human medicine practices). It is relevant to mention that most natural variants of resistance determinants arise through point mutations in target sites as well as resistance enzymes and efflux systems, affecting antibiotic binding strengths and catalytic efficiencies (Raquet et al. 1997; Crichlow et al. 1999; Nukaga et al. 2003; Rubtsova et al. 2010; King and Strynadka 2011; Sarovich et al. 2012; Ramirez et al. 2013; Kaitany et al. 2013; June et al. 2014; Shaheen et al. 2015; Mehta et al. 2015). Changes induced by mutations in the sensory/regulatory proteins that control the production of resistance determinants have also been documented (Baptista et al. 1997; DeMarco et al. 2007; Resch et al. 2008; Noguchi et al. 2004; Schmitz et al. 1998). Therefore, a summary of the sensitive target sites in bacterial cells as well as the AMR nanomachinery governing sensing of and resistance to antibiotics (Table 1) is included in the following review of fluoroquinolone and glycopeptide nanomachines.

**Table 1:** Fluoroquinolone and glycopeptide resistance nanomachines (resistance mechanisms and associated regulatory systems) in staphylococci and enterococci of animal and/or human origin

Resistance (or increased resistance) to:	Mechanism of resistance	Resistance gene(s)	Antibiotic receptor molecule, or efflux/transporter protein and (family)	Regulatory systems controlling expression of resistance genes (regulator family)	References
Fluoroquinolones	MDR active efflux/transport	*norA* *norB* *norC*	NorA (MFS) NorB (MFS) NorC (MFS)	MgrA (formerly NorR)(MarR-like) ArlSR (TCS) NorG (GntR-like)	Ubukata et al. (1989) Yoshida et al. (1990) Kaatz et al. (1991) Kaatz et al. (1993) Fournier et al. (2000) Fournier and Hooper (2000) Truong-Bolduc et al. (2003) Truong-Bolduc et al. (2003) Kaatz et al. (2005b) Truong-Boldoc et al. (2006) Truong-Boldoc and Hooper (2007) Ding et al. (2008) Santos Costa et al. (2013) Correira et al. (2017)
		*mepA*	MepA (MATE)	MepR (MarR-like)	Kaatz et al. (2005a) Kaatz et al. (2006)
		*mdeA*	MdeA (MFS)		Huang et al. (2004)
		*lmrS*	LmrS (MFS)		Floyd et al. (2010)
		*sdrM*	SdrM (MFS)		Yamada et al. (2006)
		*efmA*	EfmA (MFS)		Nishioka et al. (2009)
		*efrAB*	EfrAB (ABC transporter)		Lee et al. (2003)
	Unknown	*cfx-ofx*	Unknown		Trucksis et al. (1991)
	Gyrase protection	*qnr*-like	Qnr _E.faecalis_		Arsene and Leclercq (2007)
Glycopeptides					
Vancomycin	Types A-E, G. 1. Target modification; 2. Removal of pre-existing susceptible targets	Type A: *vanRSHAXYZ* Type B: *vanRSYWHBX* Type C: *vanCXYTRS* Type D: *vanRSYHDX* Type E: *vanEXYTRS* Type G: *vanURSYWGXYT*	VanS	VanS (S) VanR (R) (TCS)	Courvalin (2006) Depardieu et al. (2007) Wright (2011) Phillips-Jones et al. (2017a) Hughes et al. (2017) Phillips-Jones et al. (2017b)
	Target protection (cell wall thickening)	*vraTSR* *graSR* *walKR* *clpP* *stp1* *cmk* *rpoB*	Unknown	VraSR GraSR WalKR RpoB	Howden et al. (2010) Hiramatsu et al. (2014) Hu et al. (2016)
Teicoplanin and methicillin	Cell wall anchored proteins	*icaADBC,* *spa,* *sasF* *sarS*	TcaR	TcaR	Chang et al. (2013, 2014) Grove (2013)

*MDR* multidrug resistance protein, *TCS* two-compartment signal transduction system, *MFS* Major Facilitator Superfamily of membrane proteins, *MATE* Multi-Antimicrobial Extrusion protein

Van types C, E and G contain one gene for vanX and vanY peptidase and carboxypeptidase activities—in the other Types, two separate genes encode these two activities

## Fluoroquinolones

### The sensitive cellular targets

**Fig. 1:**
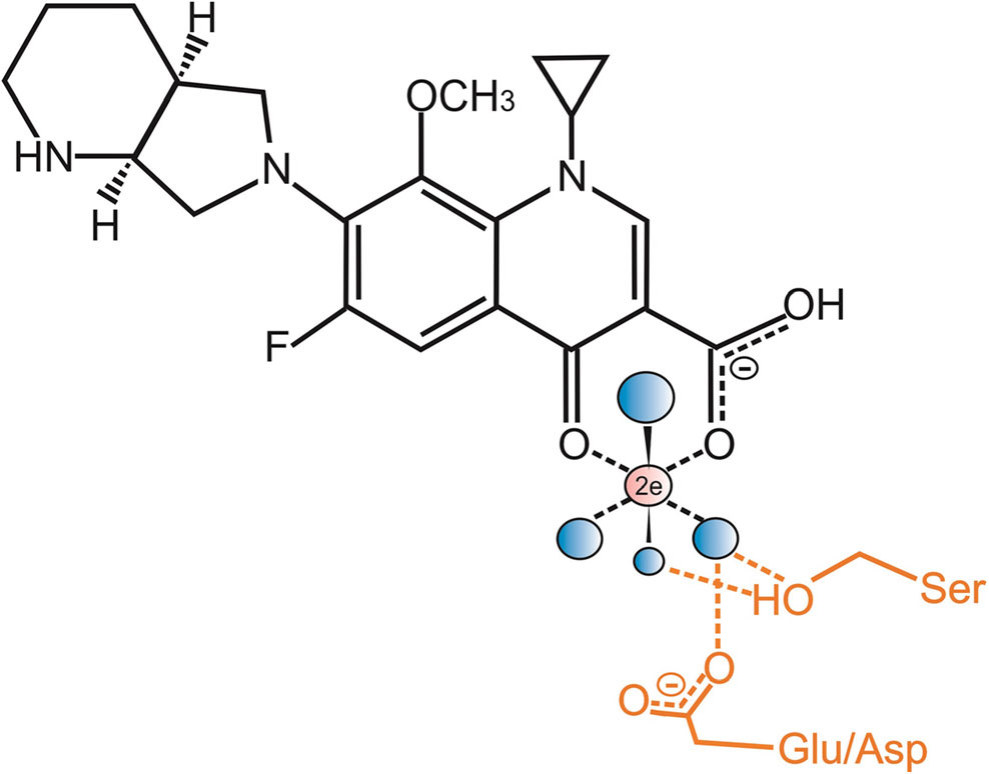
Schematic representation of moxifloxacin binding to topoisomerase IV via a water-metal ion bridge, adapted and redrawn from Aldred et al. (2014) for ciprofloxacin. For clarity, the DNA has been omitted. Moxifloxacin is shown in black; the Mg^2+^ ion that is chelated by the C3-C4 ketoacid of the antibiotic is shown in pink; the four water molecules coordinated by the Mg^2+^ ion are shown in blue. The side chains of the conserved acidic Glu88 (Asp in *Escherichia coli* and *Streptococcus pneumoniae*) and Ser84 residues of *Acinetobacter baumanii* topoisomerase IV are shown in orange, together with their hydrogen bonding to the water molecules

Fluoroquinolones (and the older generation quinolones that are currently used much less in the clinic) are used to treat infections caused by both Gram-positive and Gram-negative bacteria (Andersson and MacGowan 2003; Andriole 2005; Heeb et al. 2011; Aldred et al. 2014) (and references therein). Research in the early 1990s revealed interactions with either the A subunit of DNA gyrase or a complex of DNA gyrase and DNA (through the A subunit) to inhibit enzyme activity (Hooper and Wolfson 1991). It was later shown that another cellular target for quinolones occurs in a member of the bacterial type II topoisomerases (specifically, topoisomerase IV) as well as the gyrase (Hooper 1999, 2001; Anderson and Osheroff 2001; Drlica et al. 2008, 2009); reviewed in Aldred et al. (2014). The normal roles of these enzymes are to generate double-stranded breaks in the chromosome; DNA gyrase then introduces negative supercoils in DNA in front of the replication fork, whilst topoisomerase IV controls DNA supercoiling and is involved in the decatenation of daughter chromosomes following replication (Aldred et al. 2014; Tomašić and Mašič 2014). Quinolones bind at the interface of enzyme and DNA in the cleavage-ligation active sites and they do so non-covalently (Wohlkonig et al. 2010; Laponogov et al. 2009, 2010; Bax et al. 2010; Aldred et al. 2014). In the case of quinolone-topoisomerase binding, a water-metal ion bridge provides the link between the antibiotic and the enzyme (Fig. 1).

### Resistance determinants

**Fig. 2:**
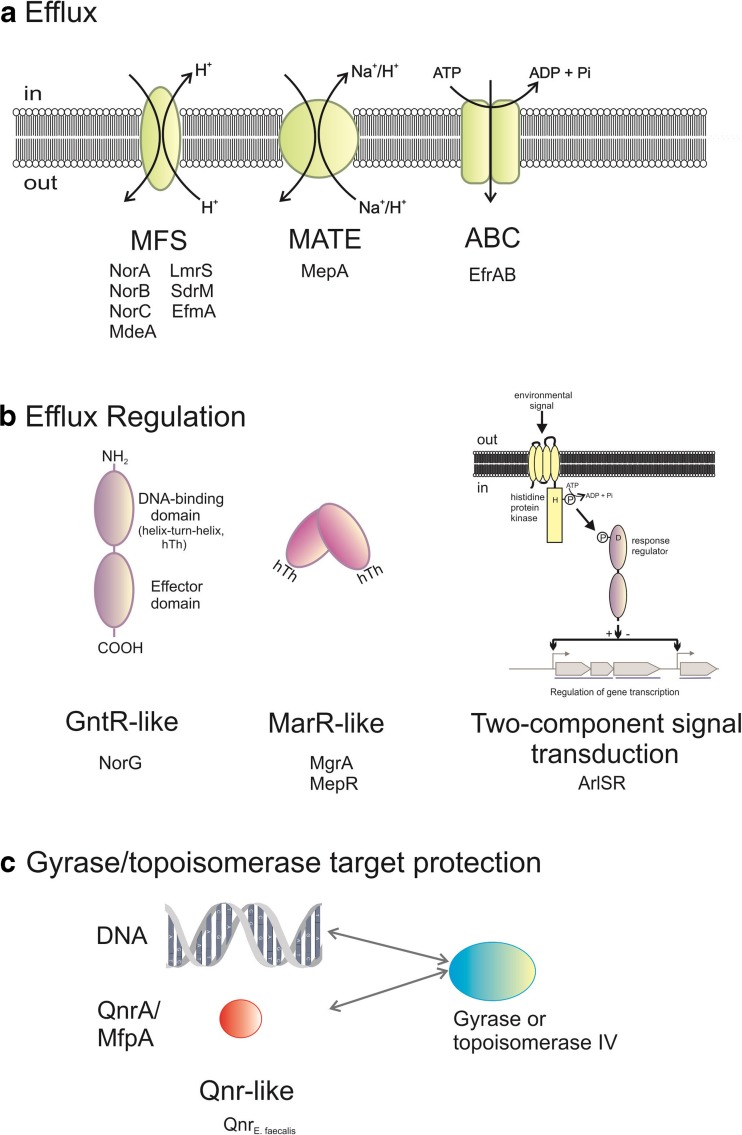
The fluoroquinolone resistance nanomachine. For full details, see text

Fluoroquinolones appear to interact with a wide range of cellular components, possibly facilitating and/or enhancing the generation of a number of mechanisms by which resistance can be mounted. Kaatz et al. (1991, 1993) first described three means by which resistance to fluoroquinolones may be generated in *Staphylococcus aureus*:

1. Mutational change in DNA gyrase, evidenced by the isolation of several point mutations in *gyrA* that confer high-level fluoroquinolone resistance (Sreedharan et al. 1990; Goswitz et al. 1992); mutations in the topoisomerases have similarly subsequently been shown to provide the bases for bacterial resistance generation (Wohlkonig et al. 2010; Aldred et al. 2012, 2013); in general, the more resistant a clinical isolate is, then the more quinolone resistance-associated mutations it contains (Komp Lindgren et al. 2003; reviewed in Jacoby 2005).
2. The *cfx*-*ofx* locus described by Trucksis et al. (1991), which confers lower-level resistance than that generated by *gyrA* mutations (Table 1); and
3. Efflux of (fluoro)quinolones from the cell by efflux pumps. In Gram-positive bacteria, the majority of efflux membrane proteins which include quinolones in their substrate profiles belong to the Major Facilitator Superfamily (MFS) of membrane transporters, e.g. NorA, NorB, NorC, MdeA, LmrS and SdrM in *S. aureus* (Table 1; Fig. 2) (Ubukata et al. 1989; Kaatz et al. 1991, 1993; Yoshida et al. 1990; Ding et al. 2008; Truong-Boldoc et al. 2006; Huang et al. 2004; Floyd et al. 2010 for LmrS efflux of gatifloxacin; Yamada et al. 2006; and reviewed in Santos Costa et al. 2013 and Correira et al. 2017) and EfmA in *Enterococcus faecium* (Nishioka et al. 2009). An efflux protein belonging to the Multiple Antibiotic and Toxin Extrusion family (MATE) includes MepA in *S. aureus* (Kaatz et al. 2005a, 2005b), whilst the ATP-binding cassette family (ABC) includes EfrAB of *Enterococcus faecalis* (Lee et al. 2003) (Table 1; Fig. 2).

Amongst Gram-positive bacteria, a further low-level resistance mechanism has been described that is plasmid-borne, Qnr _E. faecalis_. Qnr proteins resemble DNA mimics and decrease the binding of gyrase and topoisomerase IV to chromosomal DNA. This results in a reduction in the number of available enzyme targets on the bacterial chromosome. Qnr proteins also bind to the gyrase and topoisomerase IV themselves, thereby denying access for quinolones into the cleavage complexes (Fig. 2). These proteins were first discovered in Gram-negative species (reviewed in Tran and Jacoby 2002, Jacoby 2005 and Strahilevitz et al. 2009; Rodriguez-Martinez et al. 2011; and Aldred et al. 2014) but Qnr_E. faecalis_ originating from the Gram-positive bacterium *Enterococcus faecalis* was identified and characterised as a Qnr-like protein that confers intrinsic resistance to fluoroquinolones (Arsene and Leclercq 2007). It is not yet known whether or not Qnr proteins or indeed the *cfx*-*ofx* locus constitute separate independent AMR nanomachines from efflux proteins and their regulators.

### Resistance regulation determinants

Three systems that regulate expression of the *S. aureus* Nor MFS multidrug transporters have so far been described (Table 1). MgrA (formerly known as NorR) possesses a helix-turn-helix motif within a region resembling the MarR family of transcriptional regulators (Fig. 2). MgrA positively regulates *norA* expression (Truong-Bolduc et al. 2003) and negatively regulates the transcription of *norB*, a gene (*tet38*) that encodes another more selective transporter Tet38 (Truong-Boldoc et al. 2005) and *norC* (Truong-Boldoc et al. 2006). Subsequent work established that MgrA is a global regulator affecting approximately 350 genes (Luong et al. 2006) including those involved in autolytic activities and production of alpha-toxin, nuclease and protein A virulence factors (Ingavale et al. 2003; Luong et al. 2003; Truong-Boldoc et al. 2005). MgrA exhibited only weak binding to the *norB* and *tet38* promoter regions and therefore it was proposed that MgrA acts as an indirect regulator of these genes (Truong-Boldoc et al. 2005).

NorG was first identified as a transcriptional regulator of *norA* (Truong-Boldoc and Hooper 2007). NorG is a member of the GntR-like family of transcriptional regulators (Fig. 2) and it was shown to bind to the promoter regions of *norB*, *norC* and *abcA* as well as *norA* (Truong-Boldoc and Hooper 2007). It was shown to directly activate *norB* transcription but repress *abcA* (an ATP-dependent transporter of the ABC family that confers beta-lactam resistance) (Truong-Boldoc and Hooper 2007).

The third system identified as a regulator of Nor transporter expression in *S. aureus* is the ArlSR two-component signal transduction system (Fournier et al. 2000; Fournier and Hooper 2000) (Fig. 2; Table 1). Expression from the *norA* promoter was dependent on the ArlS histidine protein kinase. An *arlS* mutant which lacks ArlS exhibited increased *norA* expression (Fournier et al. 2000). Multiple putative binding sites upstream of the transcriptional start point were identified for an 18-kDa DNA-binding protein which could have been ArlR itself or another unidentified protein under ArlSR regulation; the identity of the 18-kDa protein was later shown to be MgrA (Truong-Bolduc et al. 2003; Kaatz et al. 2005b). Finally, the *arlS* mutant displayed altered growth-phase regulation of NorA confirming the role of the two-component system in *norA* expression (Fournier et al. 2000). The *arlS* phenotype also displayed changes in the ability to form biofilms, perform autolysis functions and produce peptidoglycan hydrolase, indicating the importance of the two-component system in multiple cellular functions in addition to quinolone export (Fournier and Hooper 2000).

Amongst the remaining quinolone efflux pumps listed in Table 1, the only other regulator identified so far in *S. aureus* is MepR (Fig. 2) which is a MarR-like transcriptional repressor of the MepA MATE-type multiple drug resistance pump (Kaatz et al. 2005a; Kaatz et al. 2006). MepR bound upstream of both *mepA* and its own gene *mepR* demonstrating autoregulatory activity. Repression of *mepA* expression by MepR is relieved in the presence of MepA substrates such as benzylalkonium chloride, dequalinium, ethidium bromide and pentamidine. Presumably such relief might also possibly occur using fluoroquinolone substrates, since MepA is an efflux pump with specificity for some fluoroquinolones as well as a wide range of other drugs (Kaatz et al. 2005a; Fabrega et al. 2009; Fernandez and Hancock 2012; Correira et al. 2017).

If, then, the term ‘*AMR nanomachine*’ can indeed be successfully applied to the cascade of bacterial signal sensing and transduction events required to detect fluoroquinolones and to the subsequent coupling of these detection systems to resistance machinery such as efflux pumps and other protection systems in bacterial cells, then one of the next questions is whether the same term can also be legitimately and appropriately applied to other resistance mechanisms mounted against other families of antibacterial agents. The following section addresses this question by considering the different detection and resistance mechanisms that have evolved for survival in the presence of another important and distinct family of antibacterial agents, the glycopeptides.

## Glycopeptides

Glycopeptide antibiotics have been identified as one of the highest priority classes of antimicrobial agents for risk management in clinical and agricultural settings (WHO 2011). Glycopeptide antibiotics are relatively large in size—for example, vancomycin has a molar mass of 1449 Da (Phillips-Jones et al. 2017a)—and therefore, unlike the smaller fluoroquinolones, glycopeptide drugs do not penetrate the membranes of bacterial cells and instead exert their inhibitory effects through interference with crucial bacterial processes outside the cell (Courvalin 2006). Yet bacterial cells must be able to mount resistance to glycopeptides to secure their survival (see below). But unlike the nanomachinery of fluoroquinolone resistance, an arsenal of efflux pumps will of course be redundant against antibiotics that do not enter bacterial cells. Glycopeptide resistance must be exerted by a quite different means. The question is whether the mechanisms for glycopeptide resistance and resistance regulation described below may also be considered parts of a nanomachine.

### The sensitive cellular targets

Vancomycin and teicoplanin are two important members of the glycopeptide class which are used to combat serious infections caused by Gram-positive bacteria such as bloodstream infections, infections of the skin, bones and joints, endocarditis and meningitis (Rayner and Munckhof 2005; Kristich et al. 2014; Alvarez et al. 2016). Use of vancomycin in the clinic increased markedly in the 1970s to combat methicillin-resistant *Staphylococcus aureus*. Indeed, vancomycin has become a drug of last resort to combat infections that are otherwise resistant to other front-line antibiotics. The main reasons why vancomycin is used so reservedly are as follows: (1) the toxicity of the antibiotic and (2) the poor absorption of the drug upon oral administration (Moellering 2006; Levine 2006). Vancomycin administration is carefully monitored to ensure that the concentrations are sufficiently high to be effective against Gram-positive bacterial pathogens (a serum peak level of 25–40 μg/ml and a trough serum concentration of 15–20 μg/ml, the former being equivalent to eight times the minimum inhibitory concentration), but sufficiently low as to minimise the toxic effects of the antibiotic on the patient (Tobin et al. 2002; Levine 2006; Jones 2006; Rybak et al. 2009; van Hal et al. 2013; MacDougall et al. 2016). Vancomycin is typically administered intravenously to adult patients at a starting concentration of 2.5–5.0 mg/ml (Rybak 2006). Recent biophysical investigations of vancomycin in physiologically relevant buffer conditions at both starting and therapeutic concentrations have suggested that the drug adopts two different conformations at these differing concentrations. Using sedimentation equilibrium techniques in the analytical ultracentrifuge, the SEDFIT-MSTAR algorithm and other analyses, it was shown that all the glycopeptide is dimerized at the point of clinical infusion (5 mg/ml) but at the trough serum concentration of 19 μg/ml the drug is mainly monomeric (< 20% dimerized) (Phillips-Jones et al. 2017a). Experiments employing a range of different loading concentrations were consistent with a monomer-dimer equilibrium that is completely reversible and dissociation constants indicative of relatively weak association between monomers (Phillips-Jones et al. 2017a). This is of significance because there is still relatively little understanding about the conformationally relevant form of the antibiotic during its inhibitory activity (see below).

**Fig. 3:**
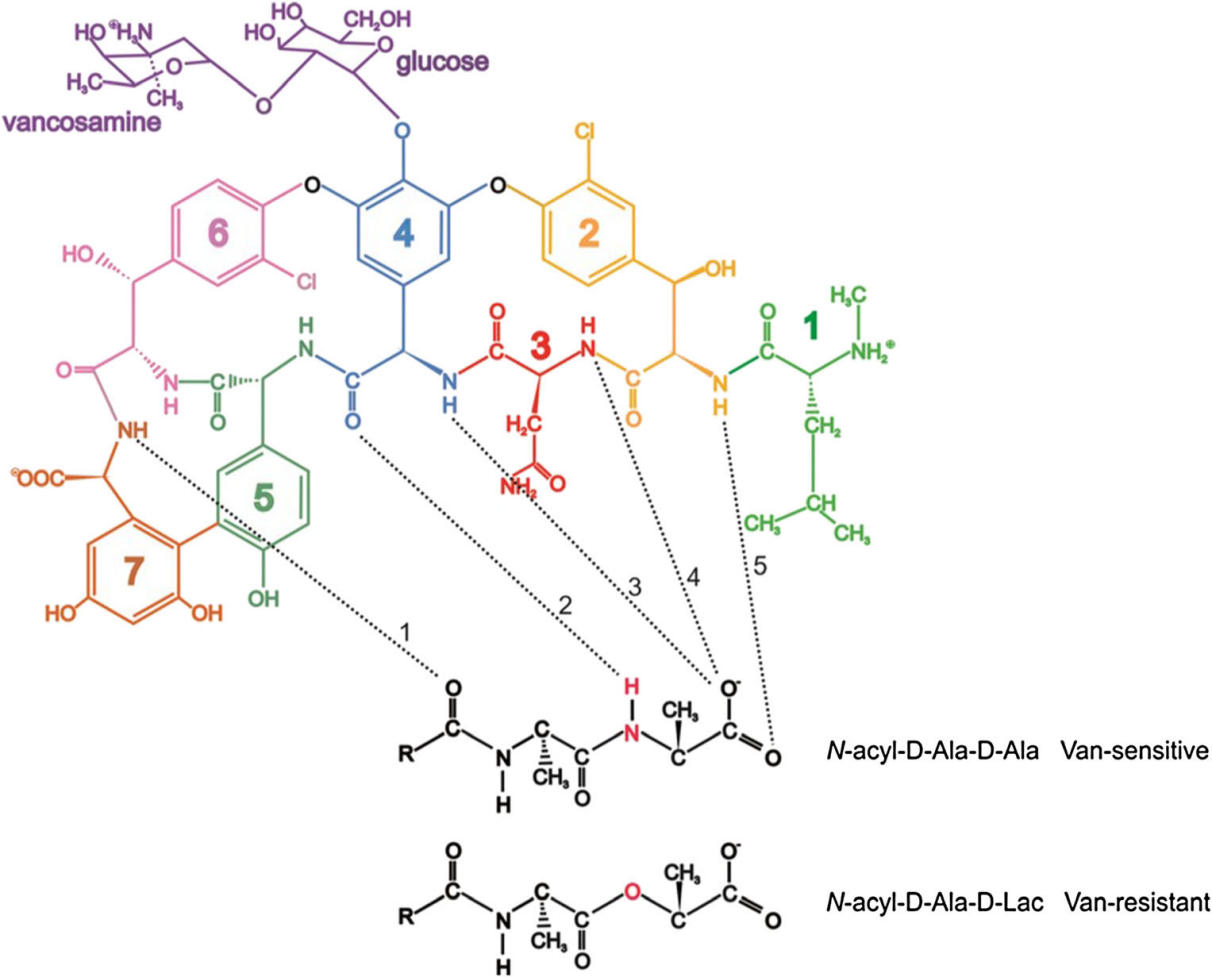
Schematic representation of vancomycin (top left) adapted and redrawn from Phillips-Jones et al. (2017a) showing the vancosamine - glucose disaccharide (purple) attached to a heptapeptide (green, N-methyl-D-leucine (residue 1); gold, *m*-chloro-β-hydroxy-D-tyrosine (residue 2); red, asparagine (residue 3); blue, D-phenyl glycine (residue 4); green-grey, *p*-hydroxy-D-phenylglycine (residue 5); pink, *m*-chloro- β-hydroxy-D-tyrosine (residue 6) and dark orange, *m,m*-dihydroxy-L-phenylglycine (residue 7)). Vancomycin binding to its sensitive target sequence (D-Ala-D-Ala) in bacterial peptidoglycan via five hydrogen bonds is shown by the dashed black lines. Hydrogen bond 2 is formed from residue 4 of vancomycin and the N-H group in D-Ala-D-Ala shown in red (middle structure). This hydrogen bond is not formed with the peptidoglycan of vancomycin-resistant bacteria that contain D-Ala-D-Lactate instead of D-Ala-D-Ala (bottom right) resulting in a total of only four hydrogen bonds for vancomycin binding which results in a 1000-fold reduced affinity of the glycopeptide for the peptidoglycan—essentially, resistance to the antibiotic

Glycopeptides such as vancomycin inhibit bacterial cell wall biosynthesis by binding to the C-terminal D-Ala-D-Ala residues of the muramyl pentapeptide of peptidoglycan precursor Lipid II (Fig. 3). Vancomycin binding results in inhibition of transpeptidase and transglycosylase activities during peptidoglycan biosynthesis, affecting crosslinking, formation of glycan chains and incorporation of peptidoglycan precursors, resulting in osmotic shock and cell lysis (Nieto and Perkins 1971; Reynolds 1989; Kahne et al. 2005; Jia et al. 2013). It has been established that for many glycopeptides, ligand binding is accompanied by the presence of asymmetric, back-to-back homodimers of the antibiotic formed through sugar-sugar recognition (see Phillips-Jones et al. 2017a for references therein). Experimental evidence shows that dimerization and binding of D-Ala-D-Ala in vitro are generally cooperative phenomena leading to the conclusion that dimerization is important for enhancing antibiotic activity (Mackay et al. 1994). Face-to-face dimers have also been reported (Mackay et al. 1994; Loll et al. 1998), as have higher order dimer-to-dimer, trimers of dimer and hexamer conformations for glycopeptide antibiotics (Loll et al. 2009; Nitanai et al. 2009), though the significance of these higher order conformations regarding inhibitory action and affinity remains to be established. There are examples of glycopeptides that do not dimerize at all; for example, lipophilic monomeric teicoplanin inserts into the membrane through the lipid moiety and it is thought to do so in such a way as to be positioned optimally for inhibitory activity (Beauregard et al. 1995; Sharman et al. 1997).

### Resistance determinants

High-level resistance to glycopeptide antibiotics was first reported amongst the enterococci in 1988 (Leclercq et al. 1988; Uttley et al. 1988) and subsequently spread to *Staphylococcus aureus* including MRSA strains (Sievert et al. 2002). Glycopeptide resistant strains of enterococci and staphylococci have spread across the world at a rapid rate (e.g. Lu et al. 2001; Iverson et al. 2002; Eisner et al. 2005; and reviewed in Schouten et al. 2000; Werner et al. 2008; Périchon and Courvalin 2009).

**Fig. 4:**
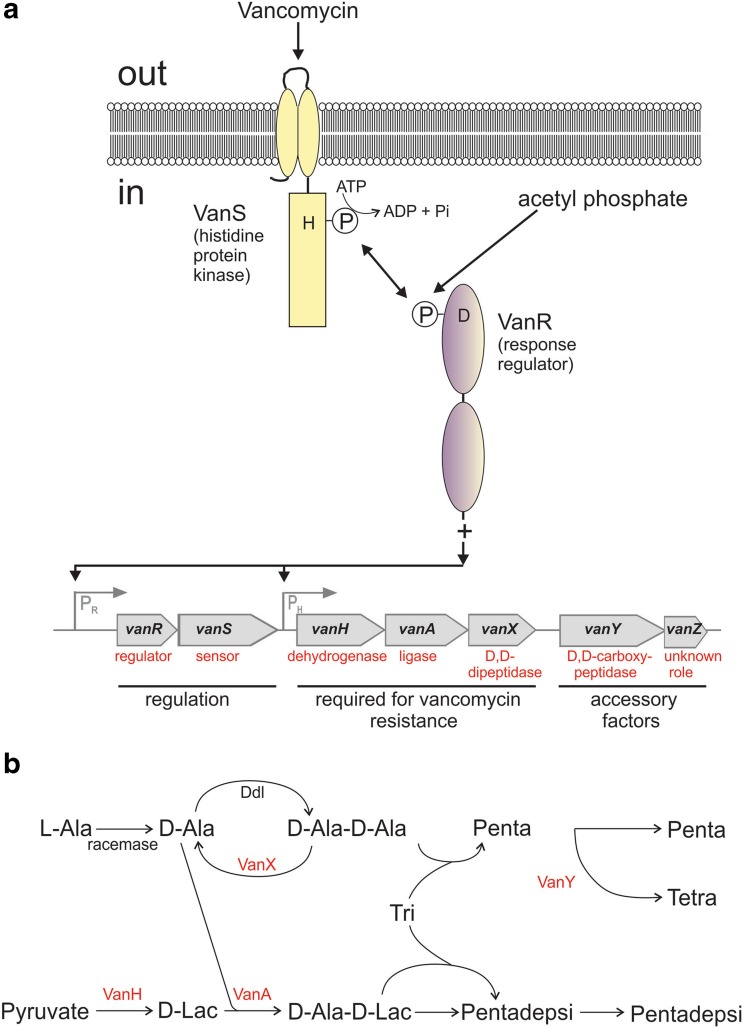
The VanA-type glycopeptide resistance nanomachine. **a** The VanS-VanR two-component signal transduction system and organisation of the *vanA* operon. Open arrows represent coding sequences and indicate the direction of transcription. The regulatory and resistance genes are cotranscribed from promoters P_R_ and P_H_, respectively; **b** synthesis of peptidoglycan precursors in a VanA-type resistant strain. Ddl, D-Ala:D-Ala ligase; penta, L-Ala-γ-D-Glu-L-Lys-D-Ala-D-Ala; Pentadepsi, L-Ala-γ-D-Glu-L-Lys-D-Ala-D-Lac; Tetra, L-Ala-γ-D-Glu-L-Lys-D-Ala; Tri, L-Ala-γ-D-Glu-L-Lys

Resistance to vancomycin occurs by two main mechanisms: (1) target modification—production of low-affinity precursors for peptidoglycan biosynthesis so that instead of D-Ala-D-Ala being incorporated into peptidoglycan monomers, other depsipeptides (D-Ala-D-lactate or D-Ala-D-serine) are synthesised and incorporated instead (Fig. 3). Vancomycin exhibits an approximately 1000-fold reduced binding affinity for D-Ala-D-Lac because of the reduced number of hydrogen bonding sites available (one crucial hydrogen bond is lost (bond 2 in Fig. 3)); and (2) removal of the high affinity precursors usually synthesised in the cell so there are no vancomycin-binding targets available (reviewed in Reynolds and Courvalin 2005; Courvalin 2006; Wright 2011). Amongst the enterococci, there are six types of resistances found (VanA-, VanB-, VanC-, VanD-, VanE- and VanG-type) which execute the above two mechanisms and these have been comprehensively described in Courvalin (2006) and Depardieu et al. (2007). Amongst *Staphylococcus aureus* isolates, only one of these types (VanA-type) has so far emerged and is thought to have been transferred from enterococci (Sievert et al. 2002; Sievert et al. 2008; Périchon and Courvalin 2009; McCallum et al. 2010). Broadly, the glycopeptide resistance nanomachine comprises (1) enzymes to synthesise the D-Ala-D-Lac or D-Ala-D-Ser depsipeptides, (2) enzymes for hydrolysis of antibiotic-‘susceptible’ peptidoglycan precursors and (3) a regulatory system to control production of these resistance enzymes. In the following sections, discussion is mainly confined to the VanA-type resistance because it is the most common type amongst clinical enterococci and the first (and, to date, only) to have disseminated to staphylococci (Table 1). The reader is referred to the review by Depardieu et al. (2007) for a detailed comparison of the genes/elements involved in each of the six vancomycin resistance types. In the VanA-type resistance, there are nine genes involved in production of transposition ability (*orf1* and *orf2*) (associated with replicative transposition when in the Tn1546 element), regulation of resistance gene expression (the *vanS* and *vanR* genes encoding the VanSR two-component regulatory system, described below), *vanH* and *vanA* encoding enzymes required for synthesis of D-Ala-D-Lac (dehydrogenase and ligase, respectively), hydrolysis of peptidoglycan precursors by a D,D-dipeptidase (encoded by *vanX*) and D,D-carboxypeptidase (*vanY*) and a ninth gene (*vanZ*) of unknown function (Fig. 4a; Courvalin 2006; Depardieu et al. 2007). This cluster of genes was originally associated with the plasmid-borne Tn1546 transposable element but are also plasmid- and chromosome-borne following horizontal gene transfer to the enterococci (Courvalin 2004; Palmer et al. 2010) and *S. aureus* (Haaber et al. 2017).

*S. aureus* possesses another mechanism of glycopeptide resistance known as ‘glycopeptide (or vancomycin) intermediate *S. aureus’* (GISA or VISA). This type of resistance was first reported in 1997 (Hiramatsu et al. 1997) and strains possessing it characteristically exhibit reduced susceptibility to glycopeptides (Hiramatsu 2001). GISA strains possess thickened peptidoglycan in their cell walls or poorly cross-linked peptidoglycan. Such conditions result in restriction of glycopeptides to the outermost layers of peptidoglycan where they quickly become sequestered by the increased numbers of free D-Ala-D-Ala target binding sites, thereby never reaching the inner layers of peptidoglycan and the crucial sites of active peptidoglycan biosynthesis (Cui et al. 2006) or at least diffuse more slowly to them (Pereira et al. 2007). Alterations and mutations in several genetic loci have been identified as responsible (Table 1) (Howden et al. 2010; Hiramatsu et al. 2014; Hu et al. 2016).

Additional resistance to teicoplanin in *S. aureus* has also been characterised (Chang et al. 2013, 2014 and refs therein). The transcriptional regulator known as teicoplanin-associated locus regulator (TcaR) belongs to the MarR family of multidrug efflux regulators (Fig. 2) (Grove 2013), involved in teicoplanin and methicillin resistance in staphylococci (Brandenberger et al. 2000).

### Resistance regulation determinants

Here we consider the A-type resistance to vancomycin only, as it is common to both enterococci and staphylococci. For regulators of other glycopeptide resistances, the reader is referred to Table 1 and Depardieu et al. (2007) and Hong et al. (2008).

The A-type *vanHAXYZ* resistance genes are regulated by the VanSR two-component signal transduction system (Arthur et al. 1992). VanS is the membrane-bound sensor kinase component involved in signal sensing and VanR is the partner response regulator component responsible for activating resistance gene expression at the P_H_ promoter (Fig. 4b) (Arthur et al. 1992; Wright et al. 1993; Holman et al. 1994; Courvalin 2006). Expression of equivalent genes of other resistance types (Types B-E and G) is also under VanSR control (Depardieu et al. 2007). Expression of the *vanSR* genes themselves is initiated from the distinct P_R_ promoter which is under autoregulatory control (Arthur et al. 1997). In the presence of glycopeptides, VanR is phosphorylated by VanS~P. VanR~P binds to the P_R_ and P_H_ promoters, promoting transcription of the *vanHAXYZ* genes and its own synthesis (Arthur et al. 1997). However, in the absence of glycopeptides (or VanS), VanR~P is still generated due to the activities of low molecular weight phosphodonors such as acetyl phosphate and/or cross-talking histidine kinases resulting in constitutive low-level activation of the P_R_ and P_H_ promoters (Fig. 4b). In the absence of glycopeptides, it is suggested that VanS serves as a phosphatase, removing phosphate from VanR through its phosphatase activity and reducing resistance gene expression in the absence of inducer. Conversely, when inducer is present, VanS transitions from phosphatase to kinase mode, resulting in increased VanR phosphorylation and elevated levels of VanR~P for induction of the *vanHAXYZ* resistance genes (Arthur et al. 1997, 1999).

The precise nature of the activating ligand for VanS has been the subject of intense interest for many years (see the comprehensive review by Hong et al. 2008). Using a variety of approaches such as reporter genes, VanX activity assays, measurements of induction of Lac-containing precursors or through detection of induced vancomycin resistance of pretreated cultures, all the evidence pointed towards vancomycin or teicoplanin as inducers of VanA-type resistance (Ulijasz et al. 1996; Arthur et al. 1999; Lai and Kirsch 1996; Mani et al. 1998; Grissom-Arnold et al. 1997; Baptista et al. 1996; Allen and Hobbs 1995; Handwerger and Kolokathis 1990; reviewed in Hong et al. 2008). However, because the structurally unrelated antibiotic moenomycin also induces VanA resistance, it was thought that the molecular effector for VanS must be a cell wall intermediate such as Lipid II which would accumulate in cells exposed to both moenomycin and vancomycin cell wall-active antibiotics (Lai and Kirsch 1996; Mani et al. 1998; Grissom-Arnold et al. 1997; Baptista et al. 1996; Allen and Hobbs 1995; Handwerger and Kolokathis 1990).

**Fig. 5:**
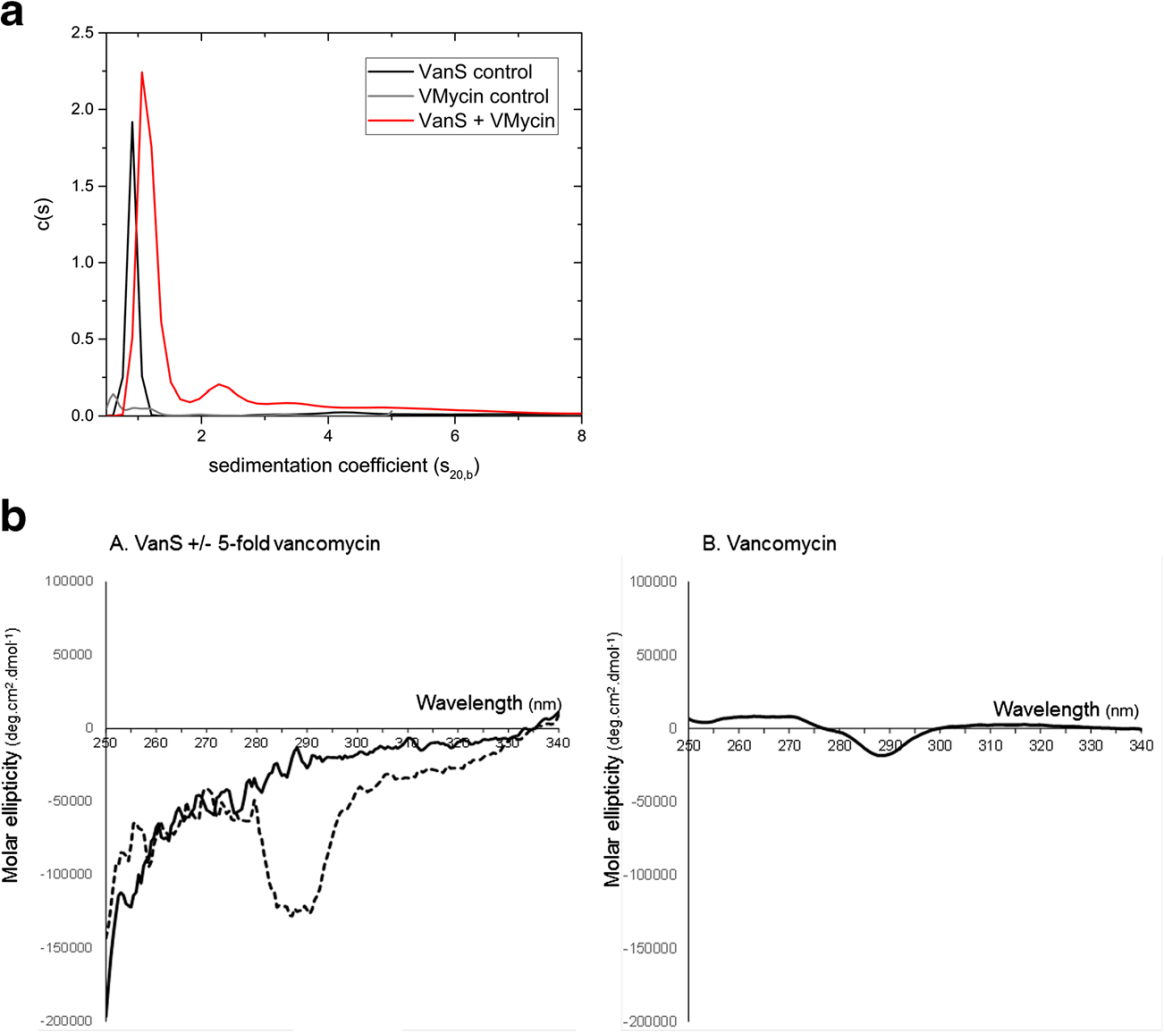
**a** Sedimentation coefficient concentration distribution, c(s) vs s_20,b_, (where s_20,b_ is the sedimentation coefficient at 20.0 ^o^C in buffer b) profile for intact VanS (5.4 μM) (black line) in HGN buffer (containing 20% glycerol) pH ~ 7.9, I = 0.1, at 20.0 °C. The rotor speed was 40,000 rpm. The profile for 12.8 μM vancomycin is shown (grey line). VanS and vancomycin is shown by the red line under the same conditions. **b** (leftmost): VanS (9 μM) difference CD spectrum (solid black line); VanS in the presence of 5-fold vancomycin (45 μM) difference spectrum (dashed black line); (rightmost): vancomycin (45 μM) difference spectrum. Reactions contained 10 mM HEPES, 20% (*v*/*v*) glycerol, 100 mM NaCl and 0.05% *n*-dodecyl-β-D-maltoside, pH 7.9. Unsmoothed data shown. Both panels reproduced with permission from Phillips-Jones et al. (2017b)

Two biophysical approaches—hydrodynamic methods in an analytical ultracentrifuge and circular dichroism spectroscopy—recently established that the purified intact VanS membrane protein from *E. faecium* B4147 interacts directly with vancomycin and teicoplanin (Phillips-Jones et al. 2017a, b) (Fig. 5). Hydrodynamic experiments in buffers containing 20% glycerol (to maintain VanS solubility) revealed that vancomycin elicits an increase in VanS sedimentation coefficient, s, of over 33% with the appearance of additional higher s components suggesting higher oligomeric forms of the receptor in the presence of the antibiotic (Fig. 5a) (Phillips-Jones et al. 2017b). These results demonstrate that VanS interacts with vancomycin. Circular dichroism measurements confirmed this finding; difference spectra obtained in the near-UV region (which interrogates changes in the tertiary structural environments of aromatic residues) for VanS alone and for VanS + 5-fold vancomycin were clearly different especially in the 280–300-nm region contributed by tyrosine and tryptophan residues (Fig. 5b) (Phillips-Jones et al. 2017b). CD-based titration experiments in the presence of detergent using increasing concentrations of vancomycin and teicoplanin revealed K_d_ values in the regions of 70 μM, and 30 and 170 μM, respectively (Hughes et al. 2017). Such K_d_ values are indicative of relatively weak binding. Weak binding may be a feasible explanation for signal transduction processes that are rapid and reversible, though it has not yet been demonstrated that the weak binding by vancomycin demonstrated in Phillips-Jones et al. (2017b) is accompanied by increased levels of VanR phosphorylation by VanS. Alternatively, the weak binding measured in these studies may reflect the absence of an essential binding accessory factor in this in vitro system, or it could reflect the absence of the natural membrane environment required for full VanS function. The latter possibility seems reasonable as VanS remained monomeric throughout all experimental conditions tested, in the presence or absence of detergent, including those associated with ligand binding which are known to induce dimerization in other sensor kinases (Phillips-Jones et al. 2017a, b). But the demonstration in these studies of vancomycin binding to VanS, albeit weakly, provides the first evidence in the clinical enterococci for the involvement of vancomycin as a molecular effector of VanA-type VanS activation. Indeed, Hughes et al. (2017) tested components of Lipid II to determine whether they too demonstrated interactions with VanS and no spectral changes were found (Hughes et al. 2017). Studies of distantly related VanS sensors in actinomycetes and VanB-type enterococci have previously provided evidence that the antibiotic itself or the antibiotic bound to the D-Ala-D-Ala substrate serves as the inducing effectors (Koteva et al. 2010; Kwun et al. 2013).

## Conclusion

Based on the above considerations of two quite different sets of resistance mechanisms, namely fluoroquinolone and glycopeptide resistances, it is clear that the enterococci and staphylococci expend significant levels of energy into ensuring survival in an antibiotic environment. There are 16 possible proteins and/or distinct complexes that have arisen amongst different strains for resistance to fluoroquinolones—though not all are likely to be present in any one individual cell (Table 1). For VanA-type glycopeptide and teicoplanin resistances, there are up to 19 proteins or complexes involved (Table 1). Therefore, in the same way that the term ‘*antibiotic resistome*’ has been used for all antibiotic resistance genes and their precursors in bacteria (Wright 2007), the term ‘*antimicrobial resistance (AMR) nanomachines*’ proposed here seems appropriate (for both fluoroquinolone and glycopeptide resistances) to reflect the large number of gene products (encoded by resistome genes) that are responsible for coordinating the sensing of these antibiotics, for transferring signal information, and for the expression of a wide variety of resistance options such as efflux pumps and efflux regulatory machinery, target protection and target modification.

Biophysical investigations have played an important role in identifying how some of the AMR nanomachines work. In this review we have focused on the use of hydrodynamic studies and circular dichroism spectroscopy to investigate the interactions between glycopeptides and the sensory receptor VanS. Such techniques are likely to prove useful for investigating the interactions between other nanomachine components in the future.
